# SARS-CoV-2 Nsp7 plays a role in cognitive dysfunction by impairing synaptic plasticity

**DOI:** 10.3389/fnins.2024.1490099

**Published:** 2024-11-21

**Authors:** Jiazheng Guo, WeiLing Li, Mengbing Huang, Jialu Qiao, Pin Wan, Yulin Yao, Lirui Ye, Ye Ding, Jianing Wang, Qian Peng, Wei Liu, Yiyuan Xia, Xiji Shu, Binlian Sun

**Affiliations:** ^1^Hubei Key Laboratory of Cognitive and Affective Disorders, Institute of Biomedical Sciences, School of Medicine, Jianghan University, Wuhan, China; ^2^Department of Immunology, School of Medicine, Jianghan University, Wuhan, China

**Keywords:** SARS-CoV-2, Nsp7, cognitive dysfunction, mitochondrial damage, synaptic plasticity

## Abstract

It has been reported that severe acute respiratory syndrome coronavirus 2 (SARS-CoV-2) infection can result in long-term neurological symptoms such as cognitive dysfunction, however the specific mechanisms underlying this phenomenon remain unclear. Initially, we confirmed a reduction in the level of synaptic proteins in SH-SY5Y neurons following SARS-CoV-2 infection. SARS-CoV-2 Nsps are crucial for the efficient replication of the virus and play important roles in the interaction between virus and host cell. Nsps screening experiments implied that Nsp7 is able to reduce the level of synapsin-1. Furthermore, overexpression of Nsp7 in SH-SY5Y cells and mouse primary neurons demonstrated that Nsp7 could decrease the levels of synaptic proteins without affecting neuronal viability. Moreover, C57BL/6 mice receiving AAV-GFP-Nsp7 injections into the ventral hippocampus displayed impaired memory ability, along with reduced dendritic spine density and synaptic protein levels. Mechanistic investigations suggested that Nsp7-induced mitochondrial damage led to ROS production and ATP levels decreasing in neurons. Additional experiments employing the ROS inhibitor NAC demonstrated that Nsp7 suppressed the expression of synaptic proteins via ROS inducing, implicating mitochondrial dysfunction in synaptic plasticity impairment and subsequent cognitive dysfunction. Our findings underscore the crucial role of SARS-CoV-2 Nsp7 in cognitive dysfunction, which is potentially mediated through impaired synaptic plasticity via mitochondrial damage. This study enhances our understanding of the pathogenic mechanisms underlying central nervous system-related symptoms associated with SARS-CoV-2 infection.

## Introduction

The emergence and global spread of SARS-CoV-2 have posed significant threats to human health. Numerous reports indicate that patients infected with SARS-CoV-2 experience a range of neurological symptoms, including ageusia, anosmia, cognitive dysfunction, and memory impairment (Premraj et al., 2022; Raveendran et al., 2021; Lechner-Scott et al., 2021). Some patients even report persistent neurological sequelae following the resolution of respiratory symptoms (Wan et al., 2021; Yasir et al., 2023). However, the pathophysiological mechanisms underlying these neurological manifestations remain incompletely understood.

SARS-CoV-2 is a single-stranded RNA virus that encodes four structural proteins (S, M, E, and N proteins) and 16 non-structural proteins (Nsp1-16; Yadav et al., 2021). While structural proteins form the virion, non-structural proteins primarily facilitate viral replication and play critical roles in virus-host interactions (Tam et al., 2023). Remarkably, SARS-CoV-2 RNA has been detected in the central nervous system (CNS) in 10/11 patients (90.9%; Stein et al., 2022). Experimental evidence from human brain organoids and transgenic mouse models expressing human ACE2 highlights the neuroinvasive potential of SARS-CoV-2, with viral RNA found in cortical neurons of COVID-19 patients (Song et al., 2021; Matschke et al., 2020). These findings suggest that SARS-CoV-2 can infect and replicate in brain cells, potentially disrupting synaptic plasticity, which is essential for cognitive functions such as learning and memory (Kourosh-Arami et al., 2023; Ladds et al., 2024; Samudyata et al., 2022; Mesci et al., 2022).

Synaptic damage, a primary contributor to cognitive dysfunction, can arise from various stimuli, including viral infections and inflammation. For example, approximately 50% of AIDS patients develop HIV-associated neurocognitive disorder (HAND), also linked to synaptic damage induced by viral proteins (Ru and Tang, 2017). SARS-CoV-2 infection disrupts cellular homeostasis by inducing autophagy, mitochondrial damage, and inflammatory pathways, with mitochondrial dysfunction being a key factor in synaptic damage. Mitochondria, present in the axon terminals and dendrites of neurons, are critical for synaptic plasticity (Cheng et al., 2010). Dysfunctional mitochondria have been implicated in various diseases, including neurodegenerative disorders like Alzheimer’s, Huntington’s, and Parkinson’s diseases (Palmer et al., 2021) and viral infections (rabies virus, hepatitis C virus; Alandijany et al., 2013; Piccoli et al., 2007) infections. Notably, SARS-CoV-2 infection is associated with mitochondrial dysfunction, characterized by decreased mitochondrial membrane potential (MMP) and increased reactive oxygen species (ROS) production (Shang et al., 2021).

Our recent investigation revealed that SARS-CoV-2 infection reduces synapse-related protein levels in neurons, with viral protein screening suggesting that Nsp7 may influence synaptic plasticity. Nsp7 and Nsp8 are critical regulators of RNA-dependent RNA polymerase (RdRP) Nsp12 (Biswal et al., 2021) and play significant roles in host-virus interactions. Nsp7 has been shown to inhibit type I and III interferon production, helping viruses evade host immune responses by targeting RIG-I/MDA5, TLR3-TRIF, and cGAS-STING signaling pathways (Deng et al., 2023). Our previous work demonstrated that SARS-CoV-2 Nsp5 can enhance cytokine production via NF-κB activation (Li et al., 2021a). Investigating the effects of non-structural proteins on neurons is crucial for understanding the mental disorders associated with SARS-CoV-2 infection.

This study focuses on the impact of Nsp7 on neurons, both *in vivo* and *in vitro*, revealing that Nsp7 may mediate mitochondrial damage, leading to decreased synaptic protein levels. Furthermore, overexpression of Nsp7 in mice correlates with cognitive dysfunction and reduced dendritic spine density. These findings enhance our understanding of the pathogenic mechanisms underlying CNS-related symptoms associated with SARS-CoV-2 infection.

## Materials and methods

### Cell culture and plasmid

The human neuroblastoma cell line SH-SY5Y was purchased from Servicebio (Wuhan, China) and cultured in minimum essential medium (MEM; Gibco, Logan, UT, United States) supplemented with an equal volume of F-12 (Ham’s F-12; Gibco, Logan, UT, United States) at a 1:1 ratio. The culture medium was further supplemented with 15% fetal bovine serum (Gibco, Logan, UT, United States) and maintained at 37°C with 5% CO2. 293 T cells and Vero E6 cells (African green monkey kidney cells) were obtained from the Chinese Academy of Science’s Cell Bank (Shanghai, China) and cultured in Dulbecco’s modified Eagle’s medium (DMEM, Gibco, Logan, UT, United States) supplemented with 10% fetal bovine serum (Gibco, Logan, UT, United States) at 37°C with 5% CO_2_.

Primary neurons were isolated from the hippocampal and cortical tissues of embryonic day 18 (E18) mice. Brain tissues of fetal mice were dissociated and dissected in 5 mL of F-12 (Gibco, Logan, UT, United States), followed by enzymatic digestion using 0.1% trypsin for 15 min at 37°C. The isolated cells were washed with PBS, diluted in DMEM/F-12 containing 10% FBS, and plated in plates precoated with 50 μg/mL poly(D-lysine). After 2 h, the medium was changed to 97% neurobasal medium (Gibco, Grand Island, NY, United States) containing 2% B27 (Gibco, Grand Island, NY, United States) and 1% glutamine (A2916801, Gibco, Grand Island, NY, United States), and the cells were cultured at 37°C with 5% CO_2_. After the cells were cultured for 3 days, the AAV solution was added, and the cells were cultured for another 3 days. Then, the cells were harvested for detection.

Fragments encoding Nsp1-10 and Nsp12-16 were cloned and inserted into pCMV-Flag vectors as previously described (Cheng et al., 2010). Lipofectamine 3,000 reagent (Invitrogen, Carlsbad, CA, United States) was used for plasmid transfection into cells according to the manufacturer’s instructions.

Adeno-associated virus (AAV) vectors used in this study were purchased from GeneChem (Shanghai, China) including AAV9-CMV-EGFP and AAV9-CMV-Nsp7-EGFP with titers of which were 2.54 E+12 v.g/ml and 3.52 E+12 v.g/ml, respectively. For overexpression of Nsp7 in primary neurons, equal amounts (2E+11 v.g) of AAV were added to 2 mL of culture medium in six-well plates. The medium was replaced after 24 h, and the cells were harvested for analysis after another 48 h of culture. For Nsp7 overexpression, AAV9-CMV-Nsp7-EGFP was bilaterally infused into the vCA1(600 nL; AP = −3.2 mm, ML = ± 3.0 mm, DV = −3.4 mm).The control group mice received infusion with AAV-CMV-EGFP.

### Packaging of lentivirus and establishment of cell lines stably expressing Nsp7

The pCDH-Flag-Nsp7-puro-EGFP and pCDH-Flag-puro-EGFP plasmids were separately cotransfected with the psPAX2 and VSV-G plasmids into 293 T cells at a ratio of 4:3:1 using Lipofectamine 2000 reagent (Invitrogen, Carlsbad, CA, United States). Four hours posttransfection, the medium was replaced with fresh culture medium, and at 48 h posttransfection, the supernatant containing lentivirus was collected through a 0.45 μm filter.

SH-SY5Y cells were plated in a 10-cm dish, 8 mL of serum-free medium was added before infection, and 1 mL of lentivirus supernatant supplemented with polybrene (Invitrogen, Carlsbad, OA, United States) was added for 2 h, followed by replacement with complete medium. On the third day postinfection, the medium was replaced with a complete culture medium containing 2 μg/mL puromycin for selection. After 1 week of selection, the stable cell lines SH-SY5Y-Flag-EGFP and SH-SY5Y-Flag-Nsp7-EGFP were successfully established.

### Antibody and reagents

Antibodies against the following proteins were purchased from the corresponding companies: TOM20 (AF1717) and LaminB1 (AF5222) from Beyotime Biotechnology (Shanghai, China); synapsin-1 (5297S), PSD95 (36233S), PSD95 (3450S), synaptophysin (25056S), synaptotagmin-1 (14558S) and NeuN (94403S) from Cell Signaling Technology (Danvers, MA, United States); Flag (20543-1-AP) and *β*-Actin (66009-1-Ig) from Proteintech (Wuhan, China); Flag (F4049) from Sigma–Aldrich (Darmstadt, Germany); SARS-CoV-2 Nsp7 (A20201) and GFAP (A0237) from ABclonal (Wuhan, China); IBA1 (ab178846) from Abcam; and SARS-CoV-2 NP (40143-MM05) from SinoBiological (Beijing, China).

N-Acetyl-L-cysteine (NAC, Cat# HY-B0215, MedChemExpress, Shanghai, China), a ROS inhibitor, was added to the cell culture at a final concentration of 2.5 mM. Mito-Tracker Red CMXRos (Mito-Tracker, C1035, Beyotime Biotechnology) was used as a mitochondrial-specific fluorescent probe at a final concentration of 200 nM.

### SARS-CoV-2 infection

The SARS-CoV-2 infection experiment followed established protocols as described previously [17]. Briefly, SH-SY5Y cells were infected with SARS-CoV-2 at a multiplicity of infection (MOI) of 1. After 1 h of incubation, the medium containing virions was removed, and the cells were washed with PBS before being cultured in MEM/F-12 medium. After 24 h, the cells were harvested and lysed for western blot analysis. All viral infection experiments were performed under biosafety level 3 (BSL-3) conditions at the Wuhan Institute of Virology, Chinese Academy of Sciences.

### Immunofluorescence

SH-SY5Y cells were plated in 24-well plates and transfected with Nsp7-Flag plasmids. After 24 h, MitoTracker was added to the medium at a final concentration of 200 nM for 25 min. Subsequently, the supernatant was removed, and the cells were fixed and then blocked with 5% BSA containing 0.1% Triton X-100 for 40 min. After blocking, the cells were incubated with a Flag antibody at a 1:200 dilution overnight. The next day, the cells were incubated with a secondary antibody, Fluor™488 donkey anti-mouse IgG (H + L; Invitrogen, Carlsbad, CA, United States), at a 1:200 dilution for 1 h at room temperature. For nuclear staining, DAPI (Beyotime Biotechnology) was used at a 1:1000 dilution. Finally, the slides were observed under a confocal microscope (Leica, TCSSP8). After the same fixation and permeabilization process described above, Vero E6 cells were incubated overnight with primary antibodies against TOM20 (Beyotime Biotechnology) and Flag (Proteintech) followed by a 1 h incubation at room temperature with the sary antibodies Fluor™488 donkey anti-mouse IgG (H + L) and Alexa Fluor™488 donkey anti-mouse IgG (H + L; Invitrogen). Subsequently, nuclear staining was performed before imaging. Confocal microscopy (Leica, TCSSP8) was used to capture images of the cells.

For tissue analysis, mice were deeply anesthetized and subjected to cardioperfusion with phosphate buffer solution, the brain was immersed in 4% paraformaldehyde for 1 day, followed by dehydration in a 30% sucrose solution for t2 days. Subsequently, the brain was frozen and sectioned with a cryotome (Leica) into 40 μm thick slices. After permeabilization and blocking, the brain tissue slices were incubated with the corresponding primary antibodies, including IBA1 (Abcam), GFAP (ABclonal) and NeuN (Cell Signaling Technology) at a 1:200 dilution overnight. Then, the slices were incubated with the appropriate secondary antibodies for 1 h, followed by staining of the nuclei with DAPI. Finally, coverslips were applied for observation and imaging under a microscope (Leica, TCSSP8).

### Western blot

Western blot was conducted following previously described methods (Zong et al., 2023). Briefly, the medium was discarded, and the cells were rinsed with PBS and then lysed with RIPA buffer (Beyotime Biotechnology) supplemented with protease and phosphatase inhibitors (Beyotime Biotechnology). The protein concentrations of the lysates were measured with a BCA kit (Beyotime Biotechnology). Subsequently, 15 μg of protein was separated by 10% SDS–PAGE and transferred onto a polyvinylidene fluoride (PVDF) membrane. The PVDF membranes were blocked with 5% skim milk and then incubated with the corresponding primary antibody (diluted 1:1000) and sary antibody (diluted 1:3000). Finally, the protein bands were visualized and visualized using a ChemiDoc XRS Gel Imaging System (Bio-Rad Laboratories, Inc., CA, United States). The gray levels of the bands were analyzed using ImageJ software (National Institutes of Health, United States), and *β*-actin was used as a loading control for normalization.

For tissue analysis, the mice were deeply anesthetized and subjected to cardioperfusion with phosphate buffer solution, the vCA1 region of the hippocampus was weighed, and the tissue was lysed and mechanically homogenized in RIPA buffer supplemented with protease and phosphatase inhibitors at a ratio of 1 mg tissue to 100 μL RIPA buffer. The proteins were quantified using a BCA Kit (Beyotime Biotechnology).

### Mitochondrial separation

SH-SY5Y cells were cultured in a 10 cm culture dish, and after 48 h of transfection, mitochondria were isolated following the instructions of the mitochondrial isolation kit (Thermo Scientific). The isolated mitochondria were lysed with RIPA buffer for western blot analysis.

### Cytoplasmic and nuclear extracts

293 T cells were cultured in a six-well plate. Following transfection with the corresponding plasmid for 48 h, cytoplasmic and nuclear proteins were extracted using the Nuclear and Cytoplasmic Protein Extraction Kit (Beyotime Biotechnology) according to the manufacturer’s instructions. Protein quantification was performed using a BCA Kit (Beyotime Biotechnology), and analysis was carried out via western blot.

### Mitochondrial membrane potential detection

The medium of cells transfected with the corresponding plasmids was replaced with a serum-free medium. The mitochondrial membrane potential was detected using Mitoprobe™ TMRM (Invitrogen, Carlsbad, CA, United States) following the manufacturer’s instructions. The stained cells were detected using flow cytometry (BD, Accuri C6 Plus), and the data were analyzed using FlowJo v10.6.2 software (Treestar, Ashland, OR, United States).

### Determination of ROS

A fluorescent MitoSox probe (M36008, Invitrogen, Carlsbad, CA, United States) was used to assess mitochondrial superoxide. The cells were replenished with serum-free medium, MitoSox Red was added at a concentration of 1.5 mM, and the cells were incubated for 30 min. ROS-containing stained cells were analyzed using flow cytometry (BD, Accuri C6 Plus), and the resulting data were processed using the corresponding software (FlowJo v10.6.2).

### ATP detection

The ATP levels in isolated mitochondria were measured utilizing an Enhanced ATP Assay Kit (Beyotime Biotechnology) following the manufacturer’s instructions. Briefly, mitochondrial proteins were extracted and quantified using a BCA kit. Subsequently, 20 μL of protein lysate and 100 μL of ATP detection working solution were added to a black opaque 96-well plate, and the luminescence (RLU) value was measured using a multifunctional plate reader (Perkin Elmer, United States). The corresponding concentration was calculated according to the standard curve.

### Electron microscopy

SH-SY5Y-Flag-EGFP and SH-SY5Y-Nsp7-EGFP cells were fixed with 2.5% glutaraldehyde (Solarbio, Beijing, Wuhan) at room temperature for 10 min, gently scraped off with a cell scraper and collected into centrifuge tubes. The cell samples were then sent to the Wuhan Institute of Virology, Chinese Academy of Sciences, for further preparation following the detailed procedures described in our previously published paper (Zong et al., 2023). The morphology of the mitochondria was observed and recorded using a 200 kV Tecnai G2 electron microscope (FEI, United States).

### Cell viability

The MTT assay (BioFroxx, Germany) was used to assess cell viability following the manufacturer’s instructions. The absorbances of the treated cells were measured at 570 nm using a microplate reader (Thermo Fisher Scientific, Inc., United States). The percentage of cell viability was calculated as (the absorbance of the Nsp7-Flag-expressing cells/the absorbance of the vector-expressing cells) × 100%.

### Animals and virus injection

Male C57BL/6 mice were raised in plastic cages with 12-h light/dark cycles and had free access to food and water at the Jianghan University Medical Animal Center. All experiments were conducted in accordance with the approved animal protocols and guidelines set by Jianghan University (approval number: JHDXLL2023-086).

Seven-week-old male C57BL/6 mice were divided into experimental groups: the experimental group receiving AAV-Nsp7-GFP injection and the control group receiving AAV-GFP injection. Mice were allowed to recover from surgery and achieve the full expression of GFP and Nsp7 over 3 weeks before behavioral testing commenced.

The injection sites and virus infection status were assessed using confocal microscopy (Leica, TCSSP8) to visualize GFP fluorescence. The sequence of behavioral tests was as follows (with at least 10 mice per group): on the first day of the fourth week, an open field test was conducted, followed by a novel object recognition test the next day. After a two-d interval, a conditioned fear test was performed. The data from the Barnes maze were obtained from the s-round mouse experiment, with 8 mice in each group, following the same procedure as described above.

### Open field test

The open field test was used to detect motor activity and anxiety in the mice. The open field test chamber was rectangular (50 × 50 × 50 cm, Xinruan, China). The mice were placed in the center area of the open field box for 10 min. The movement of the mice was recorded with video tracking software (Xinruan, China). After completion of testing on one mouse, 75% ethanol was used to reduce the smell before proceeding to the next mouse. The total distance traveled by the mice in the open field chamber, the distance traveled in the central area, and the time and number of visits to the central area were recorded.

### Novel object recognition test

The novel object recognition test (NORT) was performed to evaluate the effect of Nsp7 expressed in the hippocampus vCA1 of mice on recognition memory. For the purpose of assessing recognition, the mice were positioned in an open-field setting. The test includes two main stages: the training stage and the detection stage. During the training stage, two objects (designated 1 and 2) identical in appearance, shape and size were positioned at diagonal corners of the open field box. The touch time of the mice to the two objects was recorded using a Xinruan tracking system (Xinruan, China). When the sum of the sniffing time for the two objects was 20 s, the recording was stopped, and the mice were removed from the open field. After 1 h, we performed the detection, and one object in the open field was replaced with a novel object that was different in appearance, shape and size from the previous familiar objects. The mice were put into the open field box again, and the amount of time spent sniffing the two objects was recorded with the same strategy. The recognition index (RI) was calculated to determine the ability of the mice to recognize novel and familiar location objects. RI = (Tnovel-Tfamiliar)/(Tnovel+Tfamiliar), where Tnovel and Tfamiliar represent the sniffing time of novel and familiar objects, respectively.

### Barnes maze

To evaluate the spatial learning and memory abilities of the mice, the Barnes maze test was performed using a circular platform equipped with 20 holes, one of which contained a plastic escape box positioned underneath. Mice were motivated to find the escape box when exposed to bright light stimuli. The movement of the mice within the maze was recorded using a Xinruan tracking system (Xinruan, China). Between the experimental trials, the maze floor was cleaned with 75% ethanol to ensure consistent-testing conditions.

During the adaptation phase (day 0), each mouse was placed inside the escape box for 120 s and then allowed to explore the maze for 180 s freely. Mice that failed to locate the escape box within the allotted exploration time were allowed to remain inside for 30 s before being returned to their cages. During the acquisition phase (day 1–4), the mice were placed in the center of the circular platform, and their activities were restricted for 15 s. The mice were then permitted to explore the maze until the escape box was located within 180 s. Mice that were unable to reach the escape hole within the specified time were allowed to remain in the escape box for 30 s before being returned to their cages. This training regimen was conducted twice daily. On the exploratory trial day (day 5), the location of the escape hole remained unchanged, but the position of the escape box was altered. Each mouse was given 60 s to explore the platform freely, and the time taken to reach the escape hole, defined as the escape latency, was measured and recorded.

### Fear conditioning

Fear conditioning experiments, including auditory and situational fear experiments, were conducted to assess the memory acquisition and retrieval capabilities of the mice. The experimental mice were placed within a conditioned fear box (environment A) and allowed to explore for 180 s to acclimate to their surroundings freely. Subsequently, the mice were subjected to three pairs of auditory (15,000 Hz) and electric shock (0.75 mA) stimuli delivered via a computer program. Each stimulus pairing had an interval of 30 s, with the auditory cue lasting 30 s. In the first 2 s before the end of the sound, the mice were given a plantar-electric shock lasting 2 s. After the end of the 3 groups of sound-electric shock stimulation, the mice continued to adapt for 30 s and then stopped, and the static behavioral response was recorded. The mice were not stimulated the next day, and their behavior was recorded. On the third day, the mice were given sound cues but no other stimuli. A Freezing Scan video tracking system (Xinruan, China) was used to record and analyze freezing behavior.

### Golgi staining

The brain tissues of both the AAV-GFP and AAV-GFP-Nsp7 groups of mice were subjected to Golgi staining (FD Rapid GolgiStain™ Kit). Frozen sections with a thickness of 100 μm were utilized in accordance with the kit’s instructions, and observations were conducted under a 100× oil lens for statistical analysis. To determine the density of dendritic spines, 12 dendrites were randomly selected for each group, comprising 3 mice from the control group and 4 mice from the NSP7 expression group. The spines on each dendrite (approximately 20 μm in length) were counted, and spine density (the ratio of spine number to dendrite length) was calculated accordingly. The average density of the 12 dendrites was computed using ImageJ software.

### Statistical analysis

All the data are expressed as the mean ± standard error (mean ± SEM). The *in vitro* tests were carried out in triplicate. All the statistical analyses were performed using GraphPad Prism 6 software (GraphPad Software, Inc., San Diego, CA, United States). The statistical significance standard was set at *p* < 0.05.

## Results

### SARS-CoV-2 Nsp7 triggers a reduction in the expression levels of synaptic proteins

**Figure 1:**
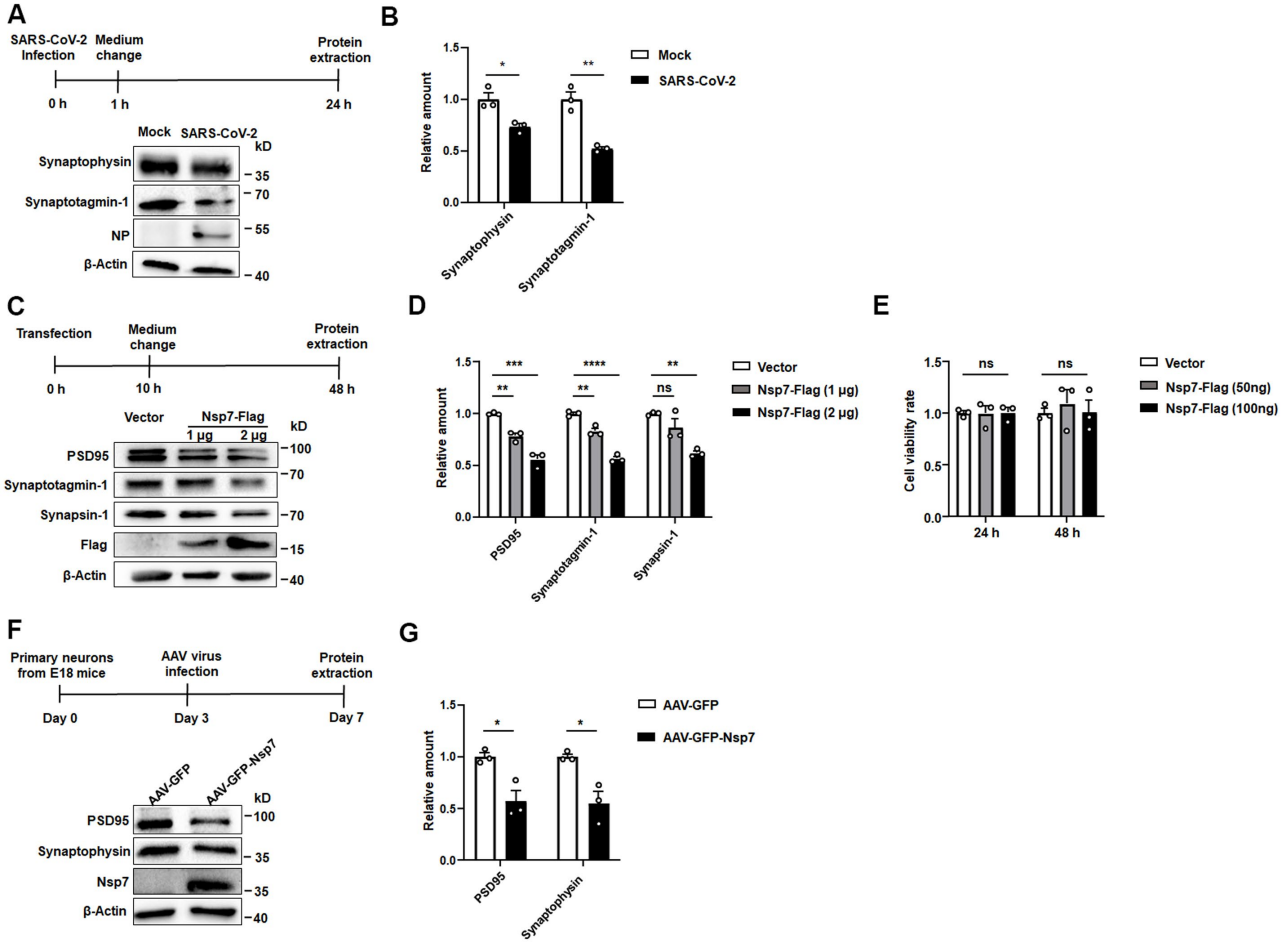
Effects of Nsp7 on synaptic protein levels in SH-SY5Y cells and primary neurons. SH-SY5Y cells were incubated with SARS-CoV-2 for 1 h at an MOI of 1. At 24 h postinfection, the levels of the synaptotagmin-1 and synaptophysin proteins, the NP protein of SARS-CoV-2 and the *β*-actin protein were detected via western blotting **(A)**, and protein expression was quantified by determining the gray level of the bands with ImageJ **(B)**. SH-SY5Y cells were transfected with 1 μg or 2 μg of the Nsp7-Flag plasmid for 48 h, and the levels of the synapsin-1, synaptotagmin-1, PSD95, Flag and β-Actin proteins were detected **(C)**, and the levels were determined **(D)**. SH-SY5Y cells were transfected with Nsp7-Flag (96-well plate, 50 ng and 100 ng per well) for 24 h or 48 h, and cell viability was measured by MTT assay **(E)**. Primary neurons were infected with AAV-GFP or AAV-GFP-Nsp7 for 3 days **(F)**, the protein levels of synaptophysin, PSD95, Nsp7-Flag and β-actin were determined and the levels were determined **(G)**. The data are expressed as the means ± SEMs and were analyzed by *t*-tests. The error bars indicate the SEMs. Asterisks indicate significant differences (*p < 0.05; **p < 0.01; ***p < 0.001; ****p < 0.0001).

It has been reported that neurons are susceptible to SARS-CoV-2 infection. Given the pivotal role of synaptic plasticity in cognitive function, we undertook a western blot analysis to evaluate the expression levels of two proteins associated with synapses in SH-SY5Y cells following SARS-CoV-2 infection. Our results demonstrated a notable decrease in the levels of synaptotagmin-1 and synaptophysin post-infection (Figures 1A,B). Synaptotagmin-1, a principal calcium sensor, is implicated in multiple stages of synaptic transmission. Synaptophysin, on the other hand, plays a role in synapse formation, maintenance, plasticity, and in modulating neurotransmitter release. Recognizing the significant function of the non-structural proteins (Nsps) of SARS-CoV-2 in virus-host interactions, we conducted a screening experiment to pinpoint which Nsp could potentially reduce the levels of synapse-associated proteins. We expressed 15 Nsps, which were previously established in our laboratory in SH-SY5Y cells (Zong et al., 2023), western blot analysis indicated that Nsp7 significantly reduced the expression level of synapsin-1 (Supplementary Figure S1), a protein essential for synaptic communication and neuronal plasticity. When SH-SY5Y cells were transfected with either 1 μg or 2 μg of the Nsp7-Flag plasmid, the western blot analysis showed a dose-dependent decrease in the protein levels of synapsin-1, synaptotagmin-1, and PSD95 (Figures 1C,D). PSD95, a marker for postsynaptic neurons, is also crucial for synapse formation, stability, and functional regulation. Additionally, we isolated and cultured primary mouse neurons and overexpressed Nsp7 using AAV. Western blot analysis revealed that Nsp7 decreased the protein levels of synaptophysin and PSD95 in these primary neurons as well (Figures 1F,G). Since cellular damage is a primary cause of synaptic injury, we assessed the viability of SH-SY5Y cells expressing Nsp7 using an MTT assay. The results indicated that Nsp7 did not impact cell viability (Figure 1E). Collectively, these findings suggest that Nsp7 lowers the levels of synaptic proteins in neurons without affecting cell viability.

### Nsp7 from SARS-CoV-2 leads to cognitive impairment in C57BL/6 mice

**Figure 2:**
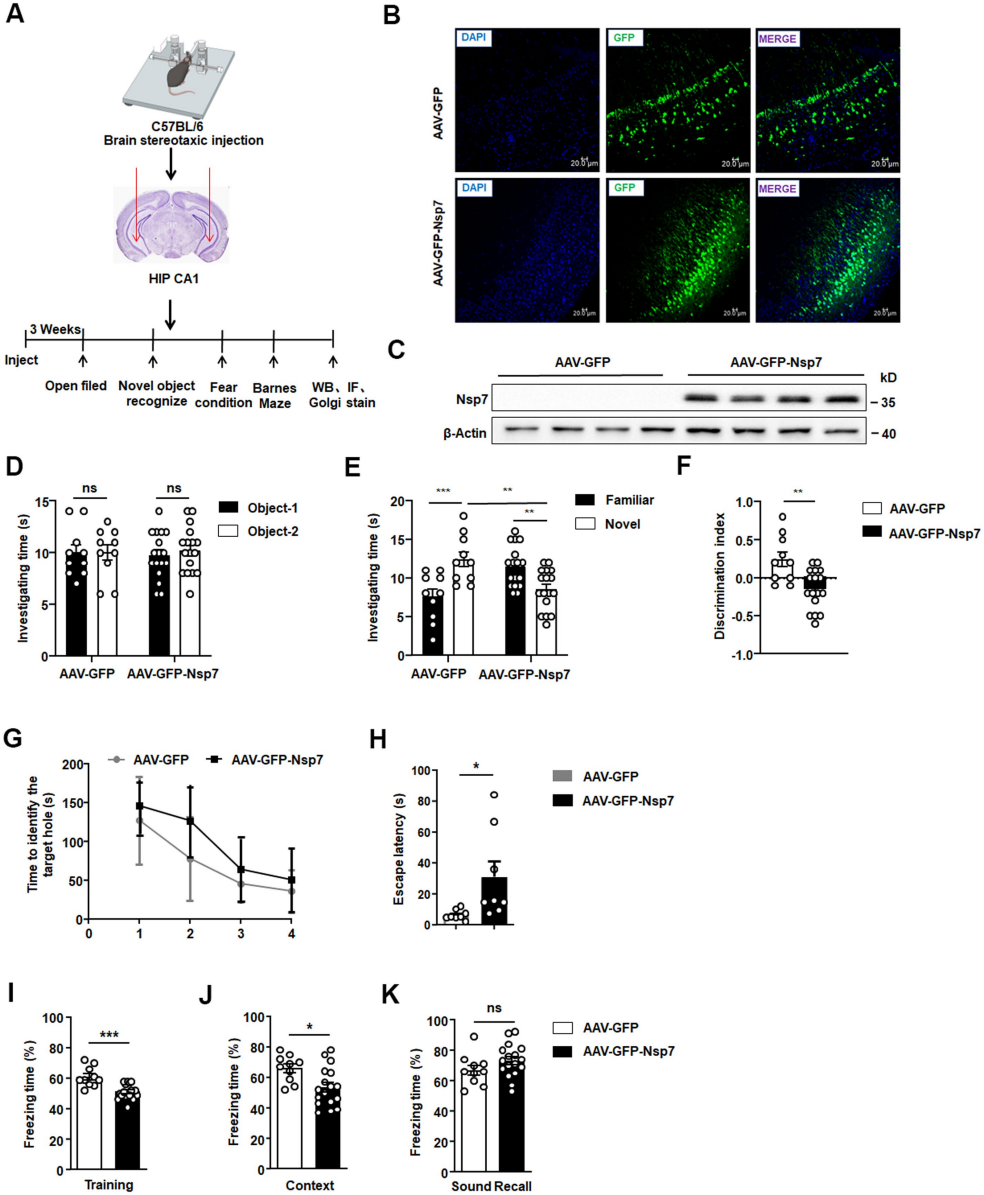
Effects of Nsp7 on the cognitive function of C57BL/6 mice. The schematic shows the virus injection and the sequences of the experiments **(A)**. Detection of the expression of AAV-GFP/AAV-GFP-Nsp7 in hippocampal tissue. The slices were stained with DAPI (blue). Scale bar = 20 μm **(B)**. **(C)** Nsp7 protein levels in the hippocampus of mice were determined by western blotting. β-Actin was used as a housekeeping control (AAV-GFP, *n* = 4 mice; AAV-GFP-Nsp7, *n* = 4 mice). The novel object recognition test was used to test the investigation time of both groups of mice during the familiarization phase **(D)** and the test stage **(E)**, and the recognition index was also calculated **(F)**. A *t-*test was performed. Mice were trained in fear conditioning. In the training stage, the freezing time rate was assayed **(G)**. The contextual cue freezing rate was assayed 24 h after the training stage **(H)**. The sound-recall freezing rate was assayed 48 h after the training stage **(I)**. Ten AAV-GFP-injected mice and 17 AAV-GFP-Nsp7-injected mice were used for the new object recognition and fear conditioning tests. Eight AAV-GFP-injected mice and 8 AAV-GFP-Nsp7-injected mice were subjected to the Barnes maze. The data are expressed as the means ± SEMs and were analyzed by *t*-tests. Two-way ANOVA was used to compare the time spent investigating the novel and familiar objects. Mice were trained on the Barnes maze for 2 trials per day for 4 days, and their performance was plotted as the time to reach the target escape hole **(J)**. The test was performed on the fifth day to determine latency **(K)**. The error bars indicate the SEMs. Asterisks indicate significant differences (*p < 0.05; **p < 0.01; ***p < 0.001).

To determine if Nsp7 diminishes the expression of synaptic proteins in mouse neurons and impacts hippocampal cognitive function, we overexpressed Nsp7 by stereotaxically injecting AAV-GFP-Nsp7 into the ventral CA1 region of the hippocampus in mice. Behavioral assessments related to learning and memory, along with Nsp7 expression, were conducted 3 weeks post-injection (Figure 2A). Initially, we validated AAV infection by observing GFP fluorescence in brain sections from four mice per group using confocal microscopy, confirming successful infection (Figure 2B). We also verified the expression of Nsp7 in the ventral hippocampal CA1 region of four mice through western blot analysis (Figure 2C). Subsequently, we conducted two rounds of behavioral experiments. The open-field test was first used to rule out any influence of Nsp7 on motor function and anxiety levels in the mice (Supplementary Figure S2), followed by three types of behavioral assessments. The novel object recognition test revealed that overexpression of Nsp7 in the ventral hippocampus did not alter the mice’s preference for two identical objects (Figure 2D) but significantly reduced their preference for new objects (Figure 2E) and the resolution index for differentiating between new and old objects (Figure 2F) compared to the control group. To assess the ability to associate memories with the environment, a fear conditioning experiment was conducted, indicating that Nsp7 also impaired memory acquisition (Figure 2G) and episodic memory retrieval (Figure 2H), without affecting auditory cue-induced memory retrieval (Figure 2I). Using mice from the second round, we evaluated spatial learning and memory abilities with the Barnes maze test. The results indicated that Nsp7 did not affect spatial learning ability (Figure 2J) but did decrease spatial memory ability in mice (Figure 2K). Collectively, these findings suggest that overexpression of Nsp7 in the ventral hippocampus diminishes the memory capabilities of mice.

### Nsp7 reduces both synaptic protein expression and dendritic spine density in mice

**Figure 3:**
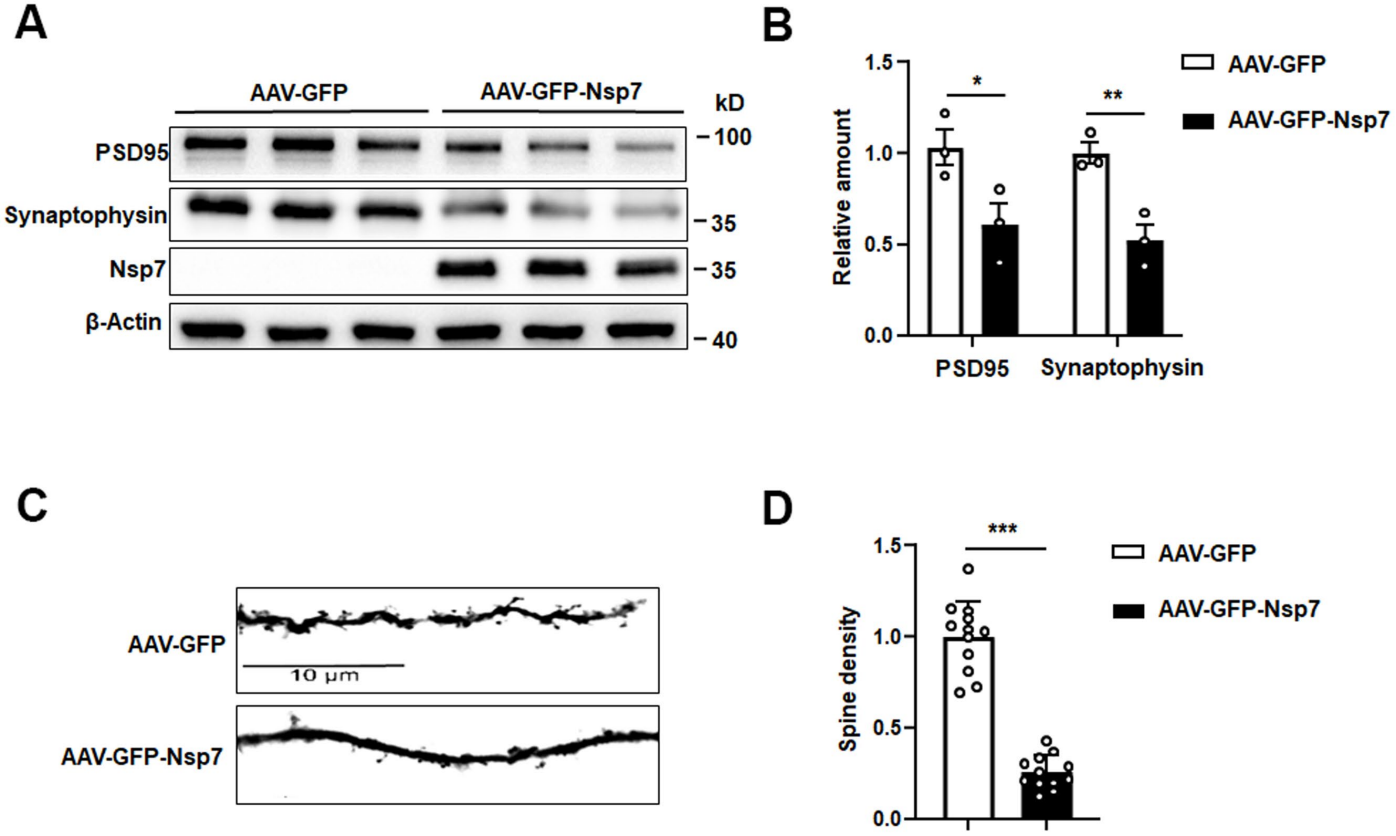
Effects of Nsp7 on hippocampal synapses. We detected PSD95 and synaptophysin proteins in hippocampal tissues from AAV-GFP and AAV-GFP-Nsp7 mice **(A)**, and the levels were determined **(B)**. The spine density was calculated from Golgi staining **(C)**. **(D)** Quantification of the dendritic spines of AAV-GFP and AAV-GFP-Nsp7 (12 dendrites derived from three to four mice per group). The data are expressed as the mean ± SEM and were analyzed by *t-*tests. Asterisks indicate significant differences (*p < 0.05, **p < 0.01, ***p < 0.001).

We further assessed the levels of synaptic proteins using western blot analysis and quantified dendritic spine density through Golgi staining in hippocampal tissues from mice. The hippocampal tissues from the Nsp7-overexpressing group showed a significant decrease in the protein levels of synaptophysin and PSD95 compared to the control group (Figures 3A,B). Moreover, Golgi staining revealed a substantial reduction in dendritic spine density in the hippocampi of Nsp7-expressing mice (Figures 3C,D). Given that dendritic spine density is directly related to the efficiency of synaptic transmission, a key process for learning and memory, our findings indicate that the synaptic deficits induced by Nsp7 in the hippocampus may underlie the observed cognitive deterioration in C57BL/6 mice.

### Nsp7 triggers mitochondrial damage

**Figure 4:**
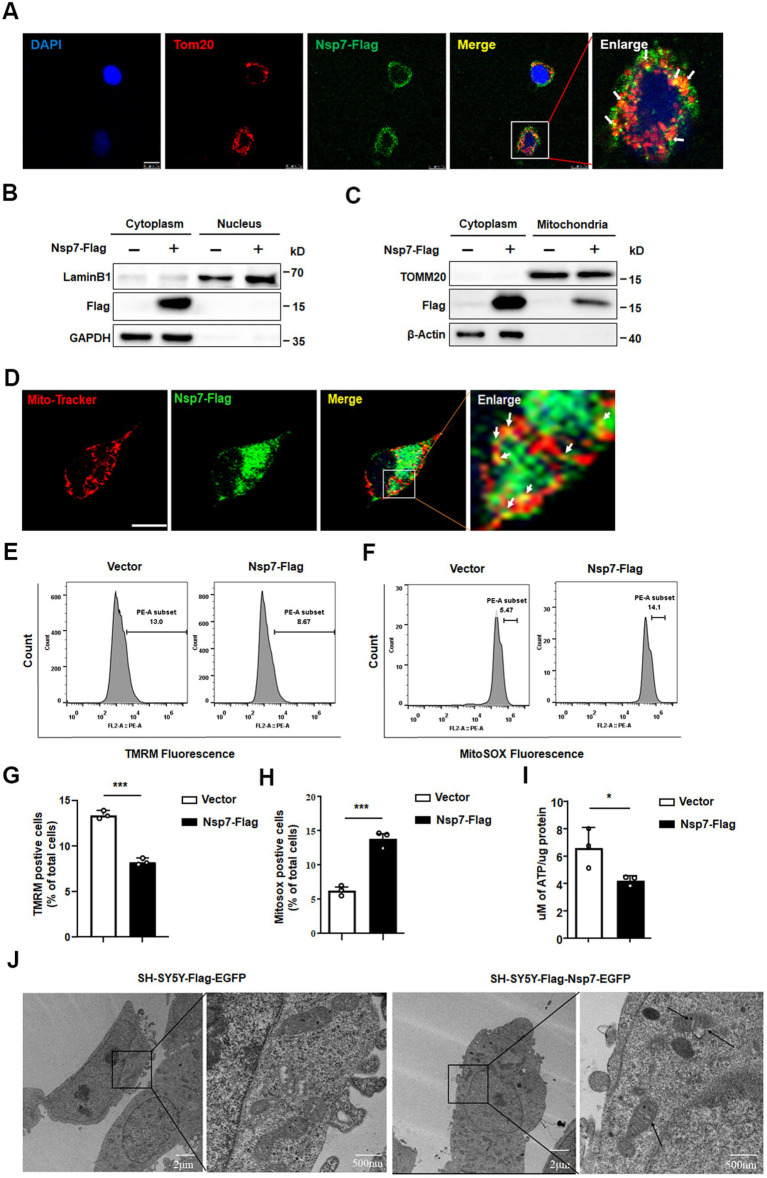
Effects of Nsp7 on mitochondria. Vero E6 cells were seeded on coverslips and transfected with 0.5 μg of the Nsp7-Flag plasmid for 24 h. The cells were immunostained with an anti-Flag antibody (green) and an anti-TOM20 antibody (red). Scale bar = 10 μm **(A)**. 293 T cells were transfected with 2 μg of empty vector or Nsp7-Flag plasmid per well in a 6-well plate. After 48 h, the cytoplasmic and nuclear proteins were fractionated and subjected to western blot analysis **(B)**. SH-SY5Y cells were transfected with 10 μg of the Nsp7-Flag plasmid in a 10 cm dish for 48 h, and cytoplasmic and mitochondrial proteins were fractionated and used for western blot analysis **(C)**. SH-SY5Y cells were transfected with 0.5 μg of the Nsp7-Flag plasmid in a 24-well plate for 24 h, incubated with MitoTracker (red) and immunostained with an anti-Flag antibody (green). The signals were analyzed via confocal microscopy. Scale bar = 10 μm **(D)**. SH-SY5Y cells were transfected with 1 μg of the Nsp7-Flag plasmid in a 12-well plate for 48 h. Then, the MMP (TMRM) **(E)** and mitochondrial ROS were detected by flow cytometry **(F)**. The relative levels of TMRM and MitoSox were analyzed with GraphPad Prism 6 **(G,H)**. SH-SY5Y cells were transfected with 2 μg of the Nsp7-Flag plasmid for 48 h, and mitochondria were extracted to detect mitochondrial ATP levels **(I)**. SH-SY5Y cells or SH-SY5Y-Nsp7 cells were analyzed by electron microscopey. Right figure is enlargement of left figure. Black arrows indicate damaged mitochondria. Scale bar = 2 μm or 500nm **(J)**. The data are expressed as the mean ± SEM and were analyzed by *t*-tests. Asterisks indicate significant differences (*p < 0.05; ***p < 0.001).

To delve into the potential mechanisms behind the synaptic toxicity caused by Nsp7, we conducted further investigations. A growing body of evidence highlights the crucial role of mitochondria in regulating axonal and dendritic spine growth, synaptogenesis, as well as the morphological and functional responses to synaptic activity (Cheng et al., 2010). SARS-CoV-2 infection has been implicated in mitochondrial damage (Stefano et al., 2021; Shang et al., 2021), and previous studies have reported the cytoplasmic localization of Nsp7 in 293 T cells (Deng et al., 2023). We overexpressed Nsp7 in Vero-E6 cells to determine its subcellular localization. The findings indicated that Nsp7 was primarily localized in the cytoplasm and showed significant colocalization with the mitochondrial marker TOM20 (Figures 4A,B). Western blot analysis confirmed the presence of Nsp7 within the mitochondria (Figure 4C), and immunofluorescence analysis revealed partial mitochondrial localization of Nsp7 in SH-SY5Y cells (Figure 4D). To evaluate the effect of Nsp7 on mitochondrial function, we measured the mitochondrial membrane potential (MMP), reactive oxygen species (ROS) levels, and ATP production. The data demonstrated that overexpression of Nsp7 in SH-SY5Y cells led to a decrease in MMP levels (Figures 4E,G) and ATP production (Figure 4I), along with an increase in ROS production (Figures 4F,H). To more clearly visualize mitochondrial damage, we established SH-SY5Y-Flag-Nsp7-EGFP stable cell lines (Supplementary Figure S3) and examined mitochondrial morphology using electron microscopy. Control SH-SY5Y cells displayed normal mitochondrial morphology under electron microscopy. In contrast, SH-SY5Y-Nsp7 cells exhibited swelling of the mitochondrial cristae and vacuolization, which are hallmarks of mitochondrial damage (Figure 4J). Collectively, these results suggest that the reduction in synaptic protein expression and dendritic spine density caused by Nsp7 may be attributed to mitochondrial damage.

### Nsp7 triggers synaptic dysfunction through mitochondrial ROS generation

**Figure 5:**
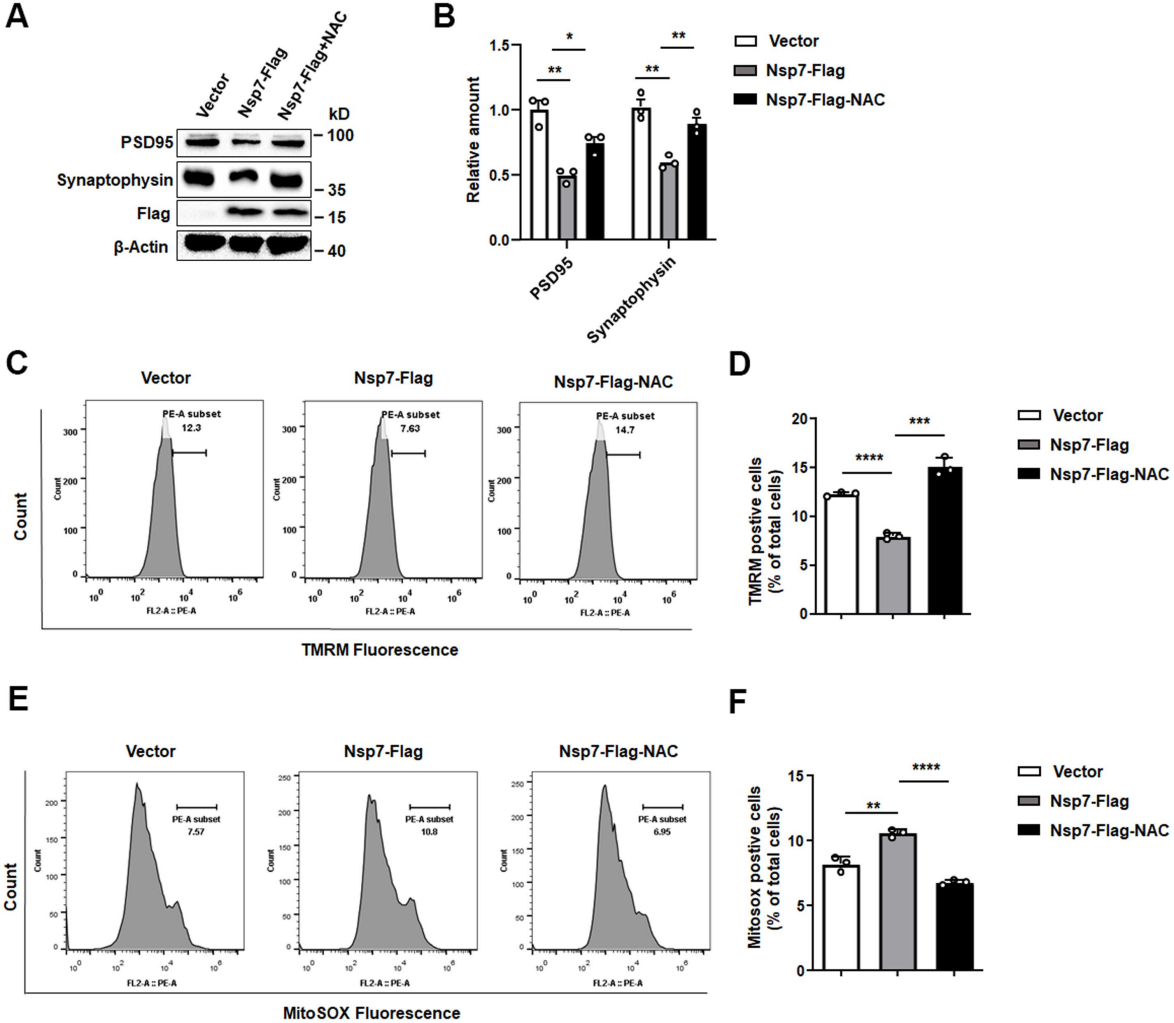
Effects of NAC on the decrease in synaptic protein expression induced by Nsp7. SH-SY5Y cells were treated with 2.5 mM NAC after 6 h of transfection with 2 μg of the Nsp7-Flag plasmid in 6-well plates. Then, PSD95 and synaptophysin were detected by western blot at 48 h after transfection **(A)**, and the levels were determined **(B)**. The same treatment was used to detect the MMP (TMRM) using flow cytometry **(C)**. The relative levels of TMRM were analyzed with GraphPad Prism **(D)**, and MitoSox was detected by flow cytometry **(E)**. The relative levels of MitoSOX were analyzed with GraphPad Prism **(F)**. All the data are presented as the means ± SEMs of triplicate values. Statistical significance was analyzed using a *t*-test. Asterisks indicate significant differences (*p < 0.05, **p < 0.01, ***p < 0.001, ****p < 0.0001).

Mitochondrial damage is known to induce ROS production, which in turn contributes to the reduction in synaptic plasticity. To further investigate the connection between Nsp7-induced mitochondrial damage and synaptic impairment, we treated SH-SY5Y cells transfected with Nsp7-Flag with the antioxidant N-acetylcysteine (NAC). We then evaluated the levels of synapse-associated proteins, mitochondrial membrane potential (MMP), and ROS. The findings showed a significant decrease of PSD95 and synaptophysin levels in cells overexpressing Nsp7, which was mitigated by prior treatment with NAC (Figures 5A,B). Additionally, the Nsp7-induced decline in MMP and the rise in ROS were substantially reversed by NAC pretreatment (Figures 5C–F). These results indicate that Nsp7 diminishes neuronal synaptic plasticity by increasing ROS production.

## Discussion

A multitude of studies has underscored the high incidence of neurocognitive impairments among individuals infected with SARS-CoV-2, highlighting the imperative to explore its neurological effects. In our research, we noted that the SARS-CoV-2 Nsp7 protein leads to a decrease in synaptic protein levels across neuronal cell lines, primary neurons, and murine brain tissue. Synapses, being essential elements of neuronal networks, are vital for the transmission of information that is fundamental to learning and memory processes, particularly within the hippocampus (Lee et al., 2021; Lisman et al., 2017). Further mechanistic investigation revealed that Nsp7 can enhance ROS and suppress ATP production by targeting mitochondria. The presence of viral proteins that cause mitochondrial damage, such as Tat protein, in HIV-associated neurodegeneration triggers, increased Ca^2+^ levels, mitochondrial ROS accumulation, and apoptosis in hippocampal neurons(Macho et al., 1999). Similarly, viruses such as HTLV-1, influenza A, and SARS-CoV-2 have been associated with central nervous system disorders, some of which involve mitochondrial dysfunction (Righetto et al., 2023). Thus, our findings also demonstrated the significance of mitochondria in the pathogenesis of COVID-19.

Given the substantial energy demands of neurons and the intricate dynamics of calcium ion and neurotransmitter release at synapses, the role of synaptic mitochondria in sustaining synaptic activity is important. Thus, maintaining a healthy mitochondrial population is imperative for optimal neuronal function. Our study revealed that some Nsp7 localized to mitochondria and was able to induce mitochondrial dysfunction, resulting in impaired synaptic plasticity. However, it is noteworthy that the impairment of synaptic plasticity in neuronal cells may not be solely due to mitochondrial damage but could also involve cellular apoptosis. SARS-CoV-2 has been implicated in various apoptotic pathways mediated by viral proteins. For instance, the spike protein has been linked to taste cell apoptosis and the release of the apoptosis-related cytokine TNF-*α* (Yamamoto et al., 2023), while it also induces apoptosis by upregulating ROS (Li et al., 2021b). Additionally, Nsp6 has been shown to induce inflammatory cell death in lung epithelial cells (Sun et al., 2022). Our screening of Nsps revealed that Nsp7 significantly decreased the expression of synaptic-related proteins but did not affect the viability of neurons.

SARS-CoV-2 has the capacity to infect three predominant types of cells within the mature brain: neurons, microglia, and astrocytes.Microglia and astrocytes are particularly important for neuronal function because they provide trophic support and contribute to shaping synaptic plasticity (Guedes et al., 2022). Studies have shown that the spike protein of SARS-CoV-2 can activate microglia, prompting them to engage in synaptic phagocytosis (Fontes-Dantas et al., 2023). Given the role of the hippocampus in cognitive functions, we used an AAV system to express Nsp7 in the hippocampus of mice to investigate its effects on neurons. Behavioral tests revealed that the overexpression of Nsp7 led to impaired memory in mice. To determine the expression of Nsp7 in brain cells, we conducted immunofluorescence colocalization experiments. The findings revealed that Nsp7 expression was significantly higher in neurons compared to microglia or astrocytes (Supplementary Figure S4). Considering the observed decrease in the levels of synapse-associated proteins, we postulate that Nsp7 may directly impede synaptic plasticity in neurons. Further cellular experiments are necessary to validate the impact of Nsp7 on microglia and astrocytes.

Our results showed that the Nsp7 protein of SARS-CoV-2 is located in the cell cytoplasm and has been reported to weaken antiviral immunity and enhance viral replication by reducing the production of IFNs (Deng et al., 2023). Interestingly, our study uncovered a novel role of Nsp7 in diminishing synaptic plasticity through mitochondrial damage, without compromising neuronal viability. This could be attributed to either the minimal cellular damage inflicted by Nsp7 or the potential activation of protective cellular mechanisms. Given the partial colocalization of Nsp7 with mitochondria, it is plausible that Nsp7 interacts with specific mitochondrial proteins, thereby disrupting their normal function.

The structural configuration of Nsp7 comprises four *α*-helices, which form an antiparallel helix bundle, namely, helices a1, a2, a3, and a4 (Zhang et al., 2021). Moreover, the sequence of Nsp7 is highly conserved among coronaviruses. To elucidate the precise mechanism underlying mitochondrial damage mediated by Nsp7, it is necessary to identify the specific protein(s) targeted by Nsp7 and delineate the key domain(s) responsible for its effects.

In conclusion, our research has demonstrated that the SARS-CoV-2 Nsp7 protein can induce cognitive impairments in mice by causing mitochondrial damage, which in turn disrupts synaptic plasticity. These findings offer valuable insights into the pathogenic mechanisms underlying the neurological symptoms triggered by SARS-CoV-2 infection.

## Data Availability

The original contributions presented in the study are included in the article/Supplementary material, further inquiries can be directed to the corresponding author.
