# Supplementary material for: SARS-CoV-2 Nsp7 plays a role in cognitive dysfunction by impairing synaptic plasticity
Source: Front Neurosci. 2024 Nov 21;18:1490099. doi: 10.3389/fnins.2024.1490099 (PMC11617585; doi:10.3389/fnins.2024.1490099)
Supplement: Supplementary file 1 [file Data_Sheet_1.docx]

**SARS-CoV-2 Nsp7 plays a role in cognitive dysfunction by impairing synaptic plasticity**

Jiazheng Guo^1,#^, WeiLing Li^1,#^, Mengbing Huang^1^, Jialu Qiao^1^, Pin Wan^1^, Yulin Yao^1^, Lirui Ye^1^, Ye Ding^1^, Jianing Wang^1^, Qian Peng^1^, Wei Liu^1^, Yiyuan Xia^1^, Xiji Shu^1,*^, Binlian Sun^1,2*^

**Supplementary Information**

**
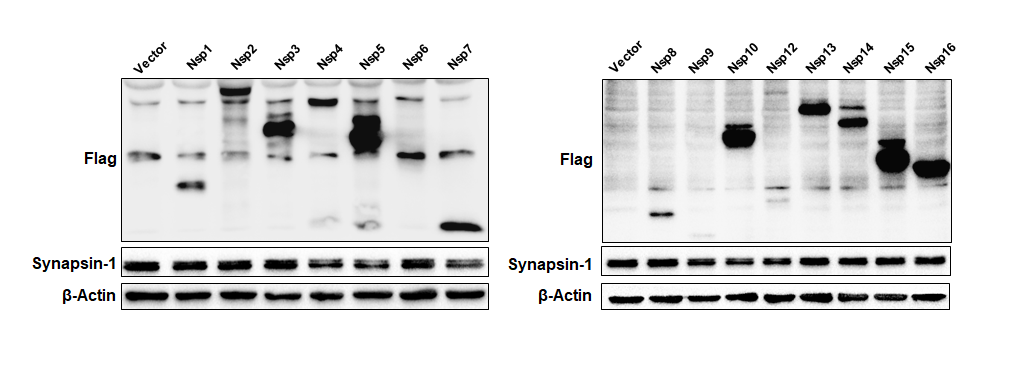
**

**Supplementary Figure 1. Non-structural protein screening.** SH-SY5Y cells were seeded in a 12-well plate, and infected with 1 μg of Flag-Nsp1 to Flag-Nsp10 and Flag-Nsp12 to Flag-Nsp16 expression plasmids. After 48 hours, the antibody to the synapsin-1, Flag and β-Actin were used to detect the corresponding proteins using western blot.

**
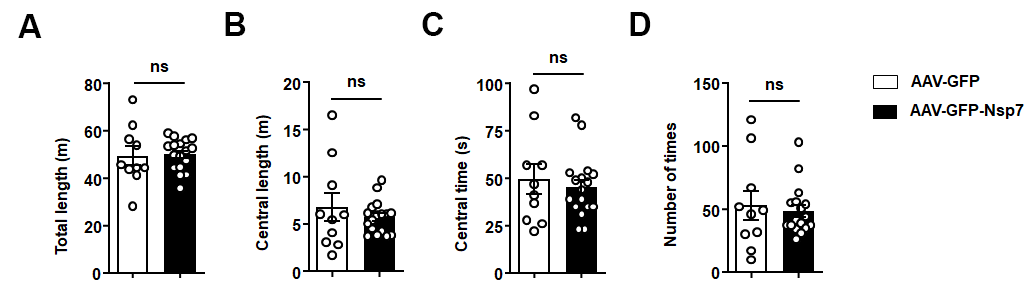
**

**Supplementary Figure 2. Nsp7 does not affect anxiety and motor ability in mice.** The open field test was used to assess the anxiety and motor ability of mice. Three weeks after the injection of AAV-GFP and AAV-GFP-Nsp7, the behaviors were recorded and the following indexes were calculated, total distance mice traveled(A), distance traveled in the central area(B), time spent in the central area(C), and number of entries into the central area(D).

**
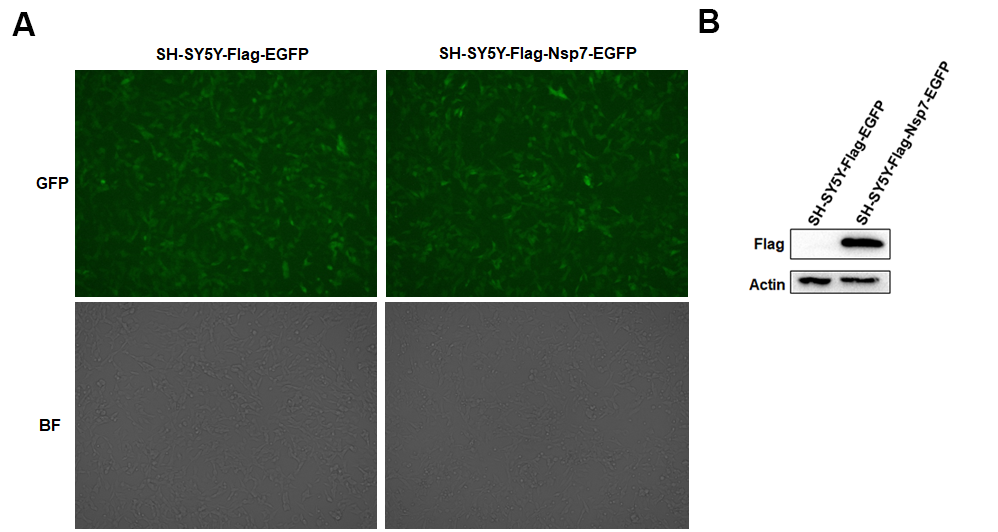
**

**Supplementary Figure 3. Establishment of Nsp7 stable expression cell lines.** (A) Observe GFP expression in cells using fluorescence microscopy. (B) SH-SY5Y-Flag-EGFP and SH-SY5Y-Flag-Nsp7-EGFP cells were collected and lysed. Flag-Nsp7, and Actin (loading control) were analyzed by Western blotting.


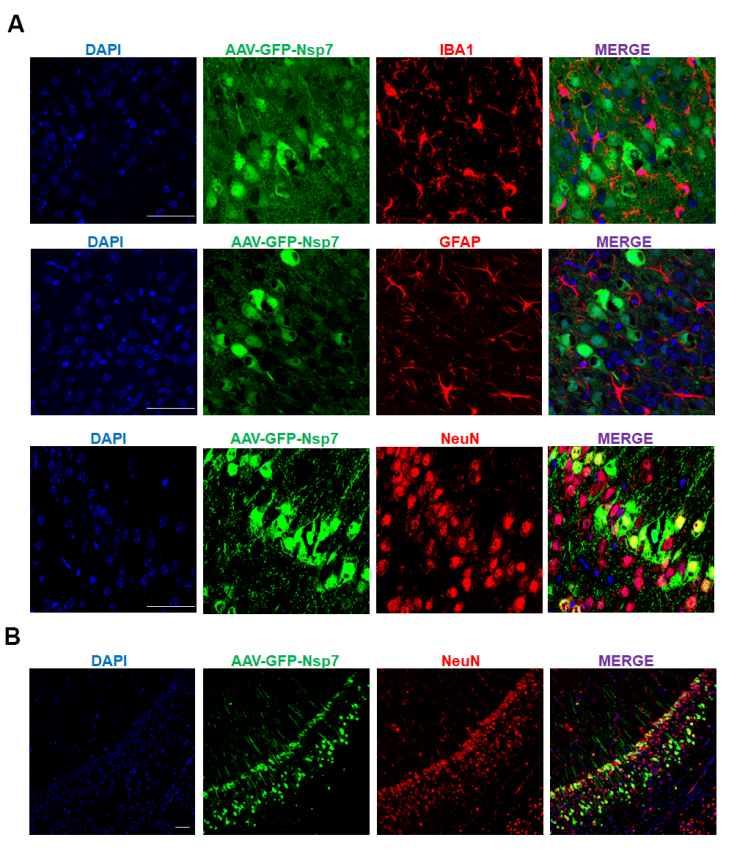


**Supplementary Figure 4. AAV-GFP-Nsp7 infects neurons.** AAV-GFP-Nsp7 was injected into the ventral CA1 (vCA1) region of the mouse hippocampus, and brain tissue slices were subjected to immunofluorescence staining. Neuronal cell marker NeuN, microglial cell marker IBA1, astrocytic cell marker GFAP, and nuclear marker DAPI were stained with corresponding antibodies. 40x magnification(A) for NeuN, IBA1 and GFAP; 20x magnification(B) for NeuN.
